# Genome Sequence of Pseudomonas veronii Strain G2, a Member of a Bacterial Consortium Capable of Polyethylene Degradation

**DOI:** 10.1128/mra.00365-22

**Published:** 2022-05-26

**Authors:** Beate Schneider, Friedhelm Pfeiffer, Mike Dyall-Smith, Hans-Jörg Kunte

**Affiliations:** a Bundesanstalt für Materialforschung und -prüfung, Division of Biodeterioration and Reference Organisms, Berlin, Germany; b Computational Biology Group, Max Planck Institute of Biochemistry, Martinsried, Germany; c Veterinary Biosciences, Faculty of Veterinary and Agricultural Sciences, University of Melbourne, Parkville, Australia; University of Maryland School of Medicine

## Abstract

Nine different bacterial isolates were recovered from landfills. Each isolate was obtained in pure culture. As a consortium, the bacteria degrade polyethylene. The complete genome sequence of strain G2 was determined by PacBio sequencing. Using the TYGS for taxonomic classification, strain G2 was assigned to the species Pseudomonas veronii.

## ANNOUNCEMENT

As part of a consortium of nine bacterial isolates, Pseudomonas veronii G2 contributes to polyethylene degradation. This strain was isolated from a former landfill site for plastic (Niemegk-Neuendorf; 52°04′59″N, 12°41′59″E) using a modified iChip procedure (1). A bacterial suspension from the landfill was diluted in agar (0.6%), loaded into an iChip, sealed with a polycarbonate membrane, deposited into soil, and incubated for 4 weeks. The emerging microcolony of strain G2 was isolated by streaking it onto agar plates, and cells from a single colony were inoculated into liquid tryptic soy broth (3 g liter^−1^, pH 7.0). After growth (24 h, 28°C), the cells were centrifuged, washed twice with phosphate-buffered saline, and kept at −80°C.

The following procedures, performed at Genewiz/Azenta (Leipzig, Germany), were previously applied to Micromonospora aurantiaca G9 (2). They included DNA extraction using the Qiagen Genomic-tip 100/G (Hilden, Germany), DNA shearing to ~10 kb using a g-TUBE device without size selection, quality (NanoDrop, pulsed field gel analysis) and quantity (Qubit v2.0) assessment, and sequencing library preparation using the SMRTbell library preparation kit v2.0 (PacBio, Menlo Park, USA). The library was sequenced on a PacBio Sequel sequencer (3) using one SMRT cell. The sequencing yielded 129,504 reads, from which 1,158,503 subreads were obtained (average length, 2,644 bp; read *N*_50_, 2,982 bp; total size, 3,063 Mbp). Within the SMRT Link Suite v5.0.0.6792, an assembly was performed using a proprietary method at Genewiz/Azenta. This included checks for quality and coverage, subread coverage reduction by random downsampling to 200-fold, and automated genome assembly using Canu v1.7 (4) with the coverage cutoff set to 40-fold. The assembly was evaluated, overlapping contigs were merged, and potential assembly artifacts were dropped. The contigs were polished with the raw subreads using Arrow. The initial assembly generated 33 contigs, with a total length of 7,111,985 bp.

A supervised assembly using Geneious v10.2 (5) for mapping the PacBio reads to the contigs with the subsequent integration and editing of the contigs resulted in four contigs. The chromosome, obtained with 409-fold average coverage (av.cov), is complete, circular, and 6,807,096 bp long, with 60.9% G+C content. There are three circular plasmids of 155,798 bp (pPVG21; 55.8% G+C content, 630-fold av.cov), 4,992 bp (pPVG22; 48.4% G+C content, 1,642-fold av.cov), and 2,150 bp (pPVG23; 58.6% G+C content, 4,133-fold av.cov). Two duplications were detected between the chromosome and pPVG21. One duplication (5,641 bp; Tn3-type transposon) was resolved by traversing the PacBio reads. The other (12,947 bp) appears to be a phage-related integrative element and was resolved by gene neighborhood comparison of the chromosomal copy with a contiguous 10-kb stretch that is found in several Pseudomonas strains, with which it shares 99% nucleotide identity. For the chromosome, the start base complies with the start base selection for Pseudomonas fluorescens Pf0-1 (GenBank accession number CP000094), which is upstream of *dnaA*. For all plasmids, the start codon of a replication protein gene was selected as the start base.

Using TYGS (6), strain G2 was assigned to the species Pseudomonas veronii, with a digital DNA-DNA hybridization (dDDH) (d_4_) value of 89.0% (95% confidence interval, 86.5 to 91.0%) (7). The low G+C content difference (0.09%) between the genome of strain G2 and the type strain, Pseudomonas veronii DSM 11331, supports this assignment. The genome was submitted to GenBank and annotated using PGAP v5.3 (8).

### Data availability.

The annotated genome has been deposited at GenBank under the BioProject accession number PRJNA788353 and the BioSample accession number SAMN23966549. The nucleotide sequence accession numbers are CP089532 (chromosome), CP089533 (pPVG21), CP089534 (pPVG22), and CP089535 (pPVG23). The Sequence Read Archive (SRA) accession number is SRX14608955.
